# Morphometric analysis of rat and mouse musculoskeletal tissues using high field MRI

**DOI:** 10.1038/s41598-025-05769-5

**Published:** 2025-07-01

**Authors:** Olivia L. Dyer, Stephanie G. Cone

**Affiliations:** Department of Biomedical Engineering, University of Delaware, 540 S College Ave, Ste 201K, Newark, DE 19716 USA

**Keywords:** MRI, Ligament, Meniscus, Rodent, Knee anatomy, Musculoskeletal system, Ligaments, Magnetic resonance imaging

## Abstract

The knee is a complex articulating joint composed of bones and fibrous connective tissues with anatomy retained across species including humans, pigs, dogs, rats, and mice. Imaging developments in high field magnetic resonance imaging (MRI) has enabled non-destructive 3D structural analysis of small animal joints to further these preclinical models. The goal of this work was to apply MRI techniques for rodent knee joints using a high field MRI scanner and to characterize the morphometry of the four primary ligaments and medial and lateral menisci. Briefly, female rat and mouse knees were imaged in a 9.4T MRI scanner and the cross-sectional area (CSA) of the ligaments and the meniscal heights and widths were recorded. Tissue dependent relationships were observed in the rat and mouse ligaments. The PCL was the largest ligament in the rats with a CSA of 0.35 ± 0.08 mm^2^, while the LCL was the largest ligament in the mice, with a CSA of 0.054 ± 0.017 mm^2^. Rat and mouse meniscal width had an anatomical location dependent relationship, while meniscal height did not. This will support future work exploring morphometric effects due to aging, injury, and disease in preclinical animal models.

## Introduction

The knee is a complex, rotating hinge joint composed of bones and fibrous connective tissues – such as ligaments, menisci, and cartilage – that provides support, enables movement, and serves as a critical component in locomotion.^1^ The anterior cruciate ligament (ACL), posterior cruciate ligament (PCL), medial collateral ligament (MCL), and lateral collateral ligament (LCL) work together to restrain the knee against excessive translations and rotations, while the medial and lateral menisci provide support and resist excessive multi-planar rotations.^1,2^ Together, these tissues provide passive stability to the knee, complementing active control through the muscle-tendon units crossing the joint. Traumatic knee injuries, such as ACL rupture, are increasingly common and are associated with pain and decreased strength and mobility during activities of daily living, resulting in an overall worsened quality of life.^3,4^ Following many serious injuries to the knee, there is an increased risk of developing post-traumatic osteoarthritis (PTOA). For example, incidence rates of PTOA after ACL rupture near 50% at 10 years post-injury.^5^ Current methods in translational research have focused on the use of animal models to study the effects of injury and to model the healing response.^6–10^.

Preclinical animal models are frequently utilized in cases where the models exhibit similar anatomical structure to the human condition. Currently, there is a wide array of animal surgical models established to understand common orthopaedic conditions.^7^ Across a wide range of animal species, including humans, pigs, dogs, rabbits, rats and mice, previous work has shown that knee joint anatomy is highly conserved.^8,11,12^ In large animal models, including pigs, researchers have studied the structure and function of native, ACL deficient, and ACL reconstructed knees to understand and quantify how ACL injury disrupts knee structure and function.^10,13–17^ In vulnerable populations, including pediatric and adolescent groups, preclinical models provide an avenue for identifying changes in the musculoskeletal system related to biological variables including sex and aging.^13,18,19^ Likewise, small animal models have proven to be a useful tool in understanding multiscale mechanical and biological changes at joint, tissue, and sub-tissue levels, as well as the ability to study pain responses and changes in behavior following knee injury.^6,20–24^ These models are also commonly implemented to understand how genetic perturbations influence the ability for native knee joint function to be maintained and restored.^21,25–27^ Previous research has shown that rodent knee joints (both mouse and rat) retain similar anatomy to humans, with comparable musculoskeletal tissue components to their human counterparts.^28,29^ These studies have previously involved gross anatomical inspection and histological analysis of the knee but lack a more comprehensive understanding of the 3D morphology and morphometry of the soft tissues in the knee – a challenge which can be addressed with recent advances in medical imaging techniques.

High field magnetic resonance imaging (MRI), or MRI with magnetic field strength greater than 3 Tesla, is an imaging modality that has been established to characterize anatomical and structural details of human and other large animal knee joints with vastly improved resolution and signal-to-noise ratios compared to conventional MRI scanners.^15,30–32^ A current limitation of conventional MRI scanning protocols is the inability to image at a sub-millimeter voxel size, which is necessary to accurately discern boundaries between soft tissues in rodent knees. Previous high field MRI work in large animal knees has shown changes in soft tissue morphometry including increasing ligament cross-sectional area (CSA) and steepening angle of orientation with growth.^18,19^ An important strength of MRI is the ability to non-invasively image the knee joint which allows for longitudinal testing. Previous work has explored how shape changes in porcine ACL bundles change during growth and the resulting changes in how the ACL carries load in the knee.^13,18^ While MRI has been employed in large animal models, typical approaches for quantifying changes in small animal models of aging and disease have been limited to destructive testing such as histology.^25,33,34^ Presently, histological analysis and gross anatomical dissection does not allow for paired biomechanical testing on the same limb due to their destructive nature. Additionally, while microCT has proven useful to quantify bone structural properties, it is limited in its ability to non-destructively image soft tissues such as muscles, ligaments, and tendons.^35–37^ Thus, there remains a need to non-destructively image rodent knee joints with appropriate resolution to ascertain 3D morphology of both the hard and soft tissues of the knee.

The objective of this work was threefold: (1) to implement a high field MRI scan sequence capable of resolving tissue anatomy in ex vivo rat and mouse knee joints, (2) to characterize the morphometric properties of the ligaments and menisci of rat and mouse knees, and (3) to quantify the relative CSA differences of rat and mouse ligaments in animals of similar skeletal maturity. It was hypothesized that (1) a 9.4T MRI with a volumetric coil would provide adequate resolution of 0.1 mm–0.05 mm isotropic voxels to resolve anatomical features of the knee, (2) all morphometric properties would have tissue dependent relationships and be larger in rats compared to mice, and (3) that the relative sizes of the ligaments would be similar across species.

## Results

### Gross anatomy of rodent knees

Gross anatomical inspection of the rat and mouse stifle joints from MRI segmentations was performed. The ligaments and menisci in both animals were found to be in similar anatomical locations, positions, and orientations, as compared to humans and other larger animals. Specifically, it was observed that the LCL originated from the lateral femoral epicondyle and attached near the proximal head of the fibula, while the MCL originated from the medial femoral epicondyle and attached on the tibia. Additionally, the PCL attached anteriorly in the femoral intercondylar notch and distally on the tibial plateau, while the ACL attached posteriorly in the femoral intercondylar notch and anteriorly on the tibial plateau. The medial and lateral menisci were observed as wedge shaped structures located between the femur and tibia.

### MRI measurement validation

**Fig. 1:**
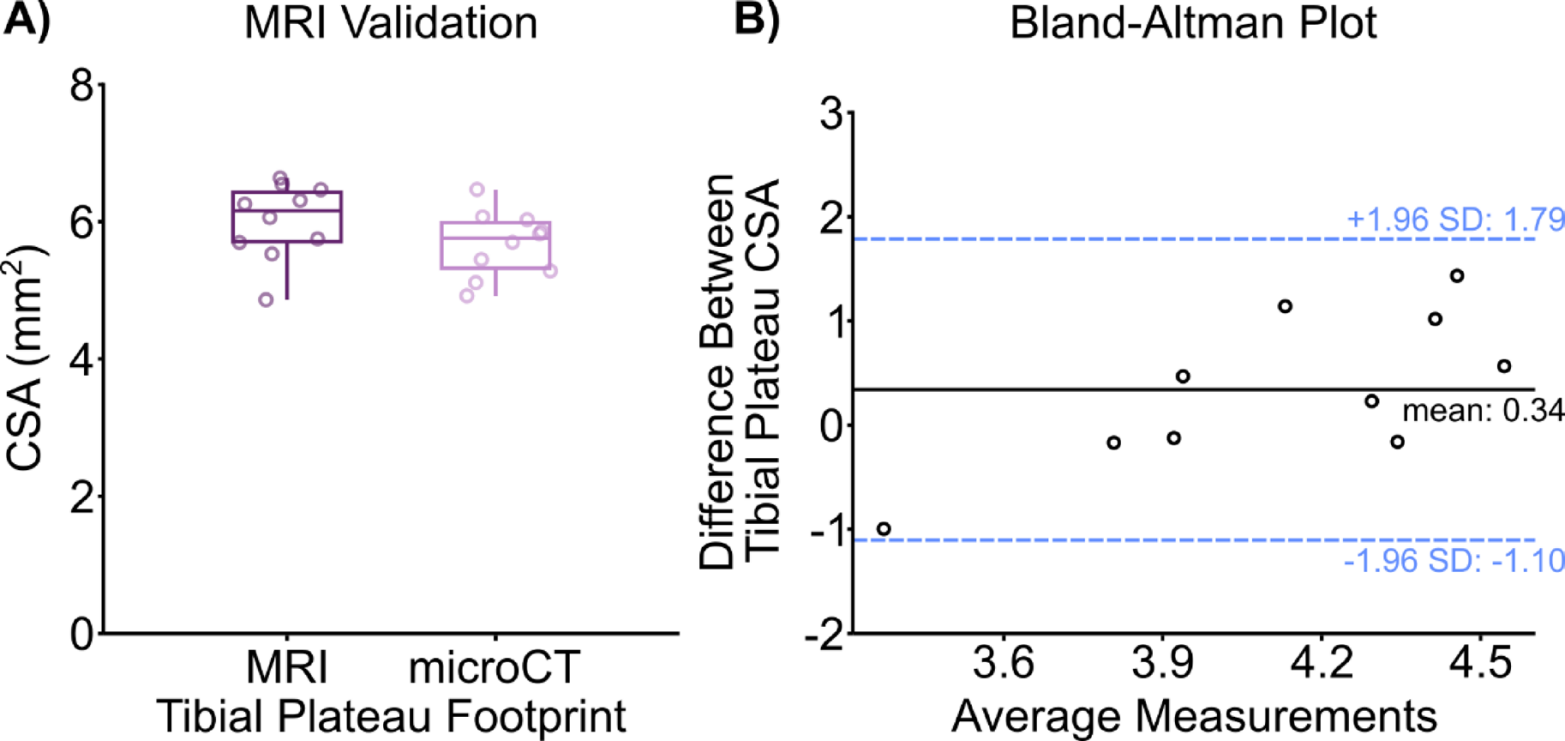
Comparison of the MRI segmentation and analysis methodology with microCT measurements between contralateral tibiae. (**A**) Cross sectional area measurements of mouse tibial plateaus that were measured with MRI and microCT. There was no significant difference between the average CSA the tibial plateaus recorded via MRI and microCT (*p* = 0.18). (**B**) Bland-Altman plot of the difference between MRI and microCT tibial plateau CSA measurements shows even scatter between high and low values of average measurements, suggesting no differences between the two imaging methodologies.

To validate the MRI segmentation and analysis measurement, the CSA of contralateral tibial plateaus in mice measured from the 3D MRI models were compared to CSA measurements recorded from microCT images (Fig. 1A). There was no significant difference in the CSA of the tibial plateau measured via MRI compared to microCT (*p* = 0.18). Additionally, the Bland-Altman plot shows no bias with increased measurement and difference between the two methodologies (Fig. 1B). Secondly, an inter class correlation (ICC) coefficient was calculated to measure the inter-rater reliability between two trained individuals in MRI segmentation. Good agreement was observed between the two individuals with the ICC value determined to be 0.79.

### Ligament cross-sectional area

**Fig. 2:**
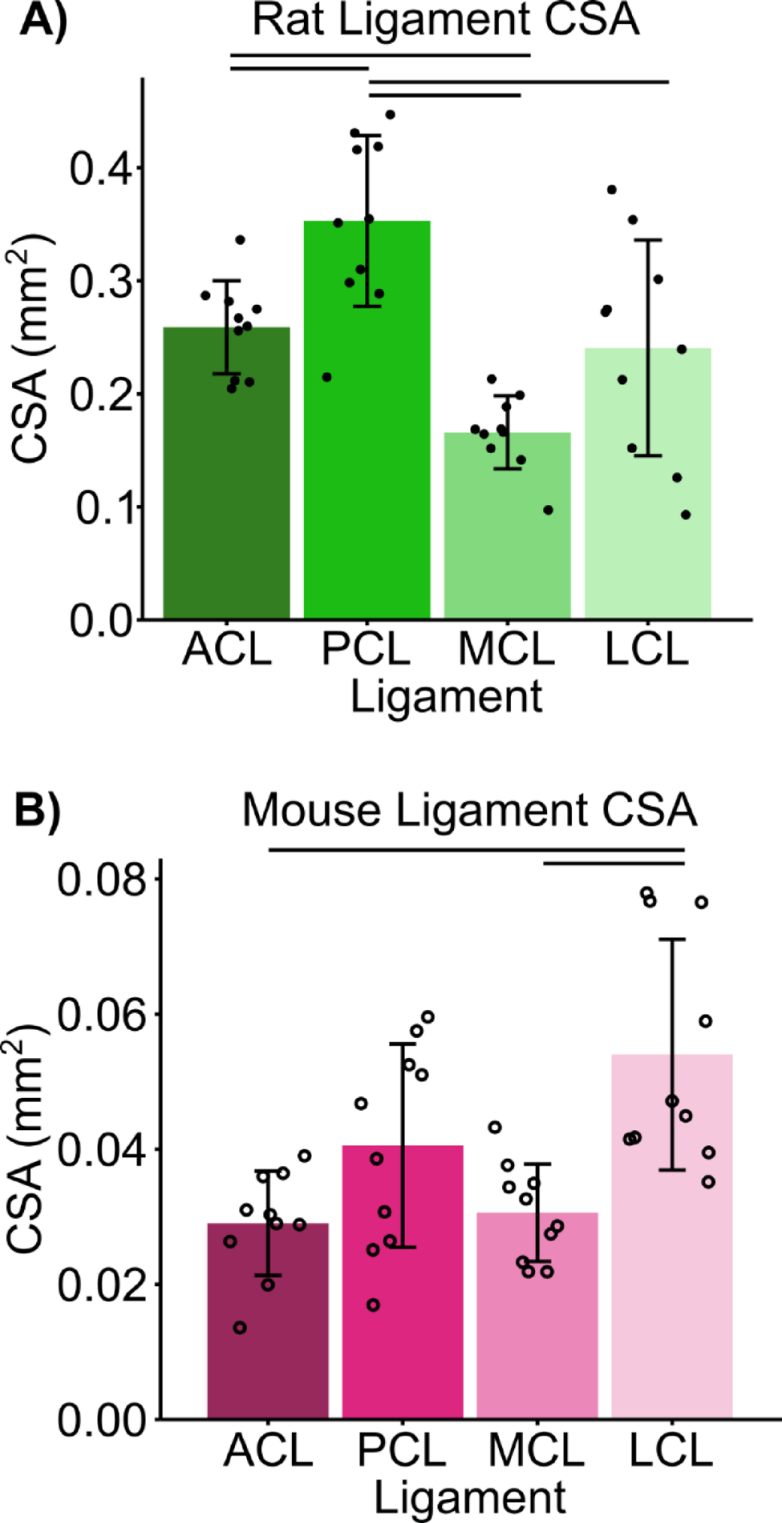
CSA is tissue dependent in (**A**) rats and (**B**) mice. There was a main effect of ligament type on CSA in (**A**) rats (H[3] = 21.8, p < < 0.001) and (**B**) mice (H[3] = 15.9, *p* = 0.001). Points represent individual specimens; error bars represent standard deviation; horizontal bars represent post hoc significant differences (*p* < 0.05).

Analysis of ligament CSA in rats and mice revealed a tissue dependent relationship, with significant differences in average CSA between ligaments within each species (Fig. 2). In rats there was a significant main effect of ligament type (H[3] = 21.8, p < < 0.001) and in mice there was a significant main effect of ligament type (H[3] = 15.9, *p* = 0.001). In rats, the PCL had the largest CSA of 0.35 mm^2^ ± 0.08 mm^2^, while in mice the ligament with the largest CSA was the LCL of 0.054 mm^2^ ± 0.005 mm^2^. In rats the LCL had the largest variability with a coefficient of variation (CV) in CSA of 40%, while in mice the PCL had the largest CV in CSA of 37%. In rats the ACL had the smallest variability with a CV in CSA of 16% and in mice the MCL had the smallest CV in CSA of 24% (Table 1). In rats the PCL was significantly larger than the other three ligaments (ACL: *p* = 0.015, MCL: p < < 0.001, LCL: *p* = 0.0029) and the ACL was significantly larger than the MCL (*p* = 0.017), but not the LCL (*p* = 0.92). In mice, the LCL was significantly larger than the ACL (p < < 0.001) and the MCL (*p* = 0.0010).

**Table 1:** Mean CSA and normalized CSA for each ligament in rats and mice. Mean meniscal heights and widths are reported at three anatomical regions for rats and mice. All data is reported as mean ± standard deviation.

Ligament Geometry					
		ACL	PCL	MCL	LCL
CSA (mm^2^**)**	Rat	0.26 ± 0.04	0.35 ± 0.08	0.17 ± 0.03	0.24 ± 0.09
	Mouse	0.029 ± 0.007	0.041 ± 0.015	0.031 ± 0.007	0.054 ± 0.017
Normalized CSA (%)	Rat	0.78 ± 0.11	1.10 ± 0.20	0.50 ± 0.10	0.71 ± 0.30
	Mouse	0.52 ± 0.15	0.71 ± 0.30	0.54 ± 0.14	0.95 ± 0.30

Meniscal Geometry				
		Anterior horn	Central	Posterior horn
Medial meniscal width (mm)	Rat	1.35 ± 0.2	0.62 ± 0.11	0.97 ± 0.15
	Mouse	0.57 ± 0.17	0.36 ± 0.07	0.48 ± 0.11
Lateral meniscal width (mm)	Rat	1.00 ± 0.2	0.79 ± 0.16	0.97 ± 0.15
	Mouse	0.42 ± 0.11	0.35 ± 0.06	0.33 ± 0.18
Medial meniscal height (mm)	Rat	0.89 ± 0.15	0.81 ± 0.12	0.88 ± 0.17
	Mouse	0.31 ± 0.07	0.29 ± 0.06	0.32 ± 0.06
Lateral meniscal height (mm)	Rat	0.75 ± 0.18	0.83 ± 0.15	0.73 ± 0.14
	Mouse	0.27 ± 0.09	0.33 ± 0.06	0.29 ± 0.05

**Fig. 3:**
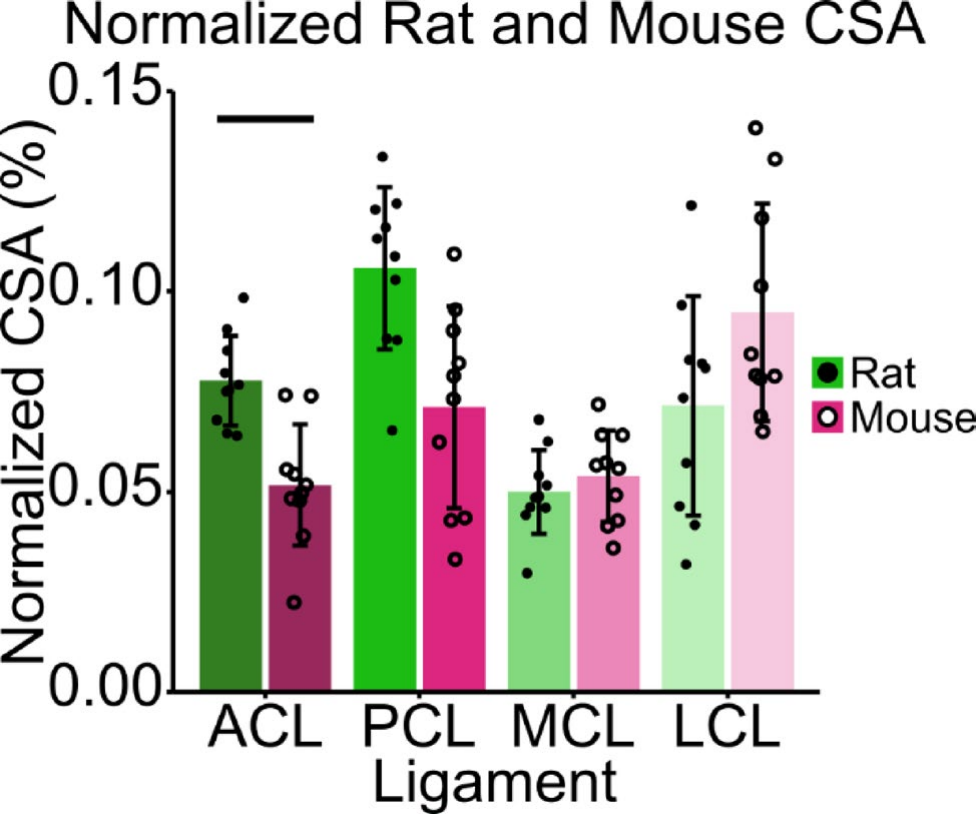
Normalized ligament CSA to tibial plateau footprint in rats and mice of similar skeletal maturity vary minimally between species. There was a significant main effect of ligament type (F[3,54] = 19.28, p < < 0.001) and an interaction between species and ligament type (F[3,54] = 11.29, p < < 0.001). The ACL was the only tissue that was significantly larger in rats compared to mice (*p* = 0.016). Points represent individual specimens; error bars represent standard deviations; horizontal bars represent post hoc significant differences between species (*p* < 0.05).

To allow for scaled comparisons between species, rat and mouse tibial plateau footprint was recorded and ligament CSA was normalized to this bone size. The average rat tibial plateau footprint was recorded as 33.35 mm^2^ ± 2.80 mm^2^, while the average mouse tibial plateau footprint was calculated as 5.67 mm^2^ ± 0.50 mm^2^. There was no significant main effect of species (F[1,18] = 2.78, *p* = 0.11) on normalized CSA, but there was a significant main effect of ligament type (F[3,54] = 19.28, p < < 0.001) on normalized CSA (Fig. 3). There was a significant interaction between species and ligament type (F[3,54] = 11.29, p < < 0.001) on normalized CSA (Fig. 3). The ACL was the only tissue that was significantly larger in rats compared to mice when normalized (*p* = 0.016). There were no significant differences between species in the remaining three ligaments, the PCL (*p* = 0.059), MCL (*p* = 0.62), and LCL (*p* = 0.46). The MCL, which had a normalized CSA averaged between species of 0.52% of tibial plateau size, was most similar between the two species (Table 1).

### Meniscal heights and widths

**Fig. 4:**
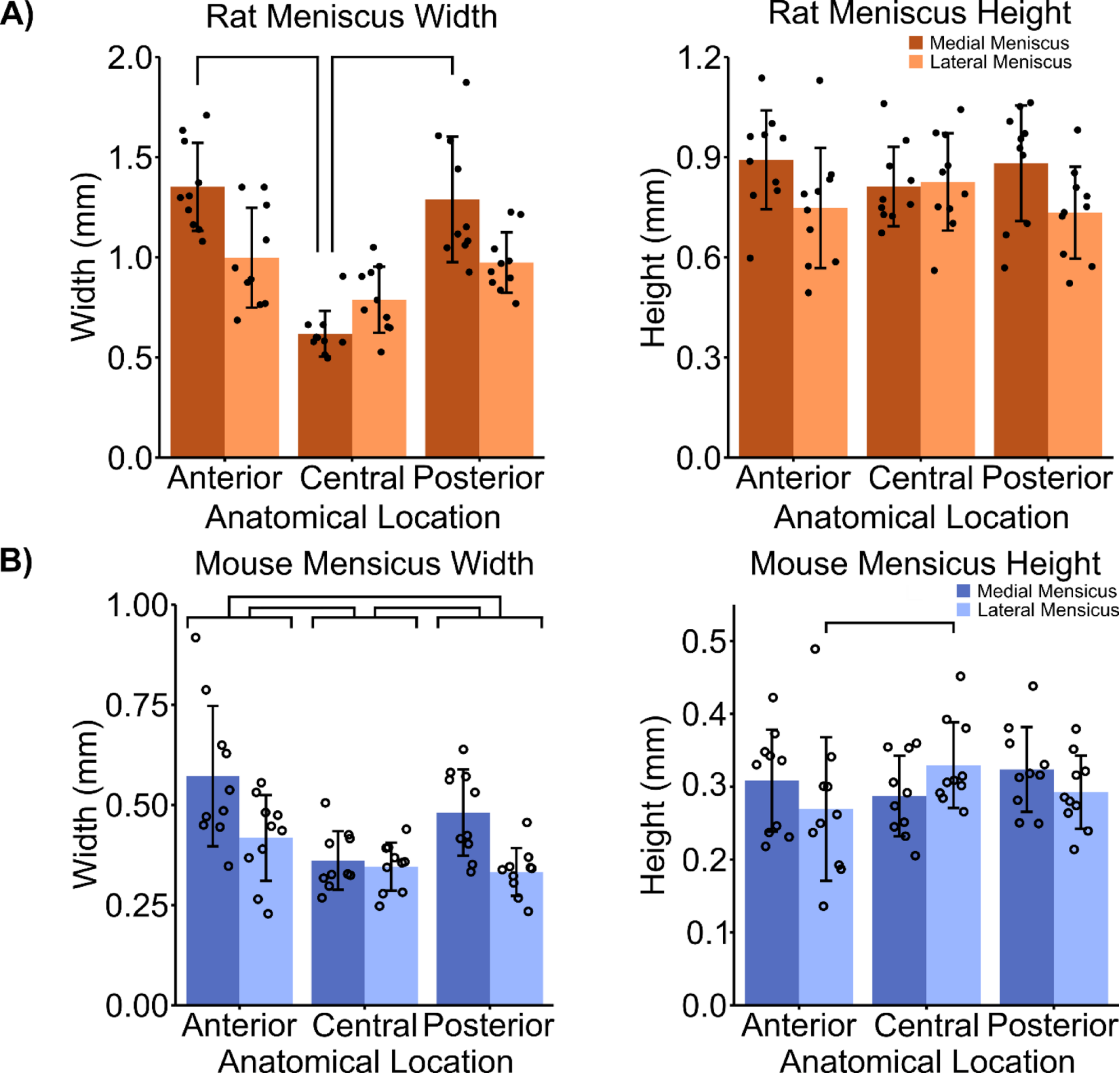
(**A**) Rat meniscal width is regionally dependent while rat meniscal height is not. There was a significant interaction of medial/lateral meniscus and anatomical location (F[2,27] = 16.8, p < < 0.001) on meniscus width. The anterior (p 0.0059) and posterior (*p* = 0.015) horns were significantly wider than the central location. There was a significant main effect of medial/lateral meniscus on meniscus height (F[1,27] = 5.43, *p* = 0.028). Specifically, the medial meniscus was significantly taller than the lateral meniscus (*p* = 0.032). (**B**) Mouse meniscal width and height are regionally dependent. There was a significant main effect of medial/lateral meniscus (F[1,27] = 18.66, p < < 0.001) and anatomical location (F[2,18] = 11.49, p < < 0.001) on meniscus width. There was a significant interaction of medial/lateral meniscus and anatomical location on meniscus height (F[2,27] = 3.81, *p* = 0.35). The anterior (p < < 0.001) and posterior (*p* = 0.044) horns were significantly wider than the central location. The anterior horn was significantly taller than the central region in the lateral meniscus (*p* = 0.029). Points represent individual specimens; error bars represent standard deviations; horizontal bars represent post hoc significant differences between tissue types or measurement regions (*p* < 0.05).

The meniscal widths for both rats and mice revealed an anatomical region dependent relationship. In rats, there was a significant main effect of medial/lateral meniscus type (F[1,27] = 12.75, *p* = 0.0014) and anatomical measurement location (F[2.18] = 37.75, p < < 0.001). Also, there was a significant interaction of medial/lateral meniscus and anatomical measurement location (F[2,27] = 16.8, p < < 0.001). In mice there was a significant main effect of medial/lateral meniscus type (F[1,27] = 18.66, p < < 0.001) and anatomical location (F[2,18] = 11.49, p < < 0.001), while there was no significant interaction between the two main effects. In rats, the anterior (*p* = 0.0059) and posterior (*p* = 0.015) horn meniscal widths were significantly larger than the widths in the central region in the medial meniscus (Fig. 4A). The rat meniscal widths averaged between medial and lateral menisci at the anterior horn, central region, and posterior horn, were 1.18 mm ± 0.3 mm, 0.70 mm ± 0.16 mm, and 1.13 mm ± 0.3 mm, respectively (Table 1). In mice, the central meniscal width was significantly smaller than the width of the anterior (p < < 0.001) and posterior (*p* = 0.044) horns. Additionally, the mouse anterior horn was significantly wider than the posterior horn (*p* = 0.02; Fig. 4B). The mouse meniscal widths averaged between medial and lateral menisci at the anterior horn, central region, and posterior horn, were 0.49 mm ± 0.17 mm, 0.35 mm ± 0.07 mm, and 0.41 mm ± 0.11 mm, respectively. In both rats and mice, the medial meniscal widths were larger than the lateral meniscal widths. In rats, the meniscal heights were not significantly different between anatomical measurement regions, but there was a main effect of medial/lateral side in rat meniscus height (F[1,27] = 5.43, *p* = 0.28). Specifically, the medial meniscus was significantly taller than the lateral meniscus (*p* = 0.038). The average rat meniscal height was 0.82 mm ± 0.16 mm and did not vary significantly across regions. In mice there were no significant main effects in meniscus height, but there was a significant interaction between medial/lateral meniscus type and anatomical location (F[2,27] = 3.81, *p* = 0.35). The average mouse meniscal height, 0.30 mm ± 0.07 mm, was comparable across regions. Only the anterior horn height was significantly shorter than the central region height in the lateral meniscus (*p* = 0.029).

## Discussion

This work aimed to expand current understanding of rodent knee joint structure by (1) utilizing high field MRI to (2) characterize gross anatomy and quantify morphometric properties of the four primary ligaments and two menisci of the rat and mouse knee joint and to (3) compare the relative differences in CSA of rat and mouse ligaments. Here, we have presented a new technique that leveraged high-field MRI to measure the size and shape of the ligaments and menisci in rat and mouse knees. There was no significant difference in mouse tibial plateau footprint measured from MRI and microCT images, suggesting that high-field MRI is a valid method that can be used to assess the geometry of rodent knees. In agreement with previous work, general inspection of the imaging data revealed that both rat and mouse knee joints retain similar anatomy to that of humans and other large animals.^7,28,38,39^ Prior studies have characterized general anatomical features of both rat and mouse knee joints using visual inspection and dissection of the knees as well as histological analysis, but were limited in their ability to quantify specific morphometric properties about the ligaments and menisci.^28,29^ Our morphometric analysis of the ligaments and menisci in both animals revealed a tissue dependent relationship in ligament CSA as well as a regionally dependent relationship in meniscus width. Additionally, our results show, unsurprisingly, that all rat morphometric features were larger than mice. Across species, we observed that the PCL and LCL had the largest cross-sectional areas, and the medial meniscus was both wider and taller at the anterior and posterior horns compared to the lateral meniscus.

In this study, both groups of animals were limited to female cohorts at a similar stage of skeletal maturity (approximately late adolescent, early adulthood), so future work is required to investigate sex and age dependent changes in rodent knee ligament and meniscus morphometry.^40,41^ Previous research in animal models revealed differences in tissue size due to sex and aging. One study in pig ACLs revealed males had slightly larger ACLs compared to age-matched females.^13,38^ Additionally, a study in mice observed sex differences in the failure force of native ACLs in mice, yet previous researchers have been limited in their ability to quantify the reason behind these observed differences.^38^ Due to the small size of rodents, imaging the soft tissues of the knee has previously not been possible, limiting the ability for past work to determine if the sex differences observed in failure force of native murine ACLs are due to the size of the tissue. Based on these findings, we expect that there will be an effect of sex and age on tissue size. Future work will include expanding the all-female cohort of rats and mice to include males of the same age and animals of varying levels of skeletal maturity to establish the effect of sex and aging on tissue factors. Lastly, a limitation of this work is the relatively long scan time (~ 90 min) and intensive data processing (i.e. manual segmentation, roughly 3 h per specimen) that could hinder application of this technique to in vivo longitudinal studies. With the recent advances in machine learning, future work could include implementing auto-segmentation techniques to help improve the time and labor required for this methodology.

The relative morphometry of the four primary ligaments, the ACL, PCL, MCL, LCL, and both menisci are retained in both rat and mouse knee joints. Importantly, lifespans of these animal models are much shorter with more rapid growth than the human case, so efforts are needed to better understand the relative age and skeletal development of the animal lifespans to human lifespans. Specimens in this study were of similar skeletal maturity – late adolescent, early adult groups – and selected due to the population relevance for many musculoskeletal injury research questions. Each of the four ligaments were found in similar positions and orientations compared to humans, large animals, and similar gross anatomical inspection work previously done in rodents.^11,28,29^ In both rats and mice, the PCL and LCL had the largest CSA, both of which provide stability against posterior translation of the tibia and varus moments about the knee.^2^ The observed variation between animals ranged from ~ 10 − 35% and is not atypical as similar levels of variation are observed in other species. For example, a study in pigs characterized ligament cross sectional area and length of tissues using a similar MRI technique presented here and found similar levels of variation that ranged from ~ 10 − 30% between animals.^19^ However, due to the small size of rodent tissue CSA, the relative amount of variation within tissues may make this methodology difficult in cases of injury, degeneration, or ageing where there may not be a large enough change in CSA to detect a significant difference in tissue size between groups.

Preclinical rodent models are common translational tools used to elucidate key components in the progression and prevention of post traumatic osteoarthritis (PTOA), yet there is little information about the morphometry of the soft tissues found in rodent knee models. Due to the prevalence of debilitating ACL injuries with serious long-term consequences including increased risk of re-rupture and the development of PTOA, the ACL is one of the most commonly studied ligaments in the knee.^11,12^ Past work has relied on gross anatomical characterization and histology to understand the composition and orientation of the ACL and the other ligaments and menisci in the knee. While these techniques can provide information on composition and orientations of these structures, they are limited in their ability to accurately quantify 3D measurements of these tissues. To our knowledge no prior study has quantified the 3D morphometry of the rat and mouse ACL and remaining ligaments and menisci in situ using MRI.^28,29,33,38^ In addition to measuring rat and mouse ligament morphometry, we compared normalized ligament CSAs to measure how these structures might be similar or different across species. Ligament CSA was normalized to tibial plateau footprint in accordance with past work in a pig model that compared changes in ligament size and shape across skeletal development. This method of normalization was selected as it is a commonly used metric in MRI imaging methodology.^13,19^ We found that across species tissue size was relatively similar, with only the ACL being significantly larger in rats compared to mice.

Finally, we identified and characterized healthy meniscus geometry – heights and widths at three anatomically relevant regions – in rats and mice. A regionally dependent relationship in meniscal width was observed while no spatial relationship was observed in meniscal height. On average, the medial meniscus was wider and taller than the lateral meniscus in both rats and mice. Previous work that characterized rodent gait found that ground reaction forces were oriented medially and pushed toward the midline.^42^ A larger medial meniscus could help to resist the additional medial forces through knee joint, due to the wide step width observed in rat gait.^42^ In large animal studies (e.g. human, pig, and sheep), a similar regional dependent relationship in meniscal width has been demonstrated.^43^ However, in contrast to a previous large animal study, both the rats and mice did not have a regional dependent relationship in meniscal height.^43^ In our work, the height of the anterior location was significantly taller than the central location in the lateral meniscus in mice. In rats the average medial meniscus height was significantly larger than the lateral meniscus. While these differences were observed, there were minimal trends observed in the heights of the menisci in both rats and mice, with heights being relatively consistent across anatomical locations. Previous imaging studies of rodent joints have shown ossification in the outer portions of the menisci, which could account for some of the variability in reported meniscus heights and widths.^28,29,44^.

Innovations in high field MRI enable ongoing efforts to explore the growth and development of rodent knee joints. Previous work in large animal models has shown there is tissue specific growth, with the ACL and LCL experiencing allometric growth, while the patellar tendon and MCL experience isometric growth.^19^ It remains unclear whether similar growth trends through skeletal development will be observed in rats and mice. In addition to a deeper understanding of tissue level structural changes in the knee joint during growth and development, this work provides a foundation for future work to explore how injury and disease change tissue structure. Given that rodents, and mice in particular, are a common preclinical model, the ability to non-destructively image the soft tissues of the knee is critical to elucidate the structure-function relationships of these soft tissues. Unfortunately, the best methods of preventing PTOA disease progression after knee injury remain unclear, motivating future studies of the healing response of the knee following injury to improve treatment strategies and reduce the risk of further damage and degeneration of the joint.^45,46^.

In this work we presented a novel MRI scan sequence to resolve the individual tissues in the knee, allowing for future work to explore how tissue geometries change due to sex, aging, injury, and disease. We observed significant differences in ligament size in both rats and mice and identified a regionally dependent relationship in meniscus width but not height.

## Materials and methods

### Sample preparation

Thirty stifle joints were dissected post-mortem at the hip joint from female, 20-week-old Long Evans rats (*n* = 10, Charles River Laboratory, Wilmington, MA) and female, 12-week-old C57BL/6 mice (*n* = 20 contralateral limbs, Jackson Laboratories, Bar Harbor, ME). Tissue was kept hydrated throughout dissection with phosphate buffered saline (PBS). Specimens for this ex vivo study were collected postmortem from IACUC approved research studies at the University of Delaware in accordance with relevant guidelines and regulations. No in vivo testing was performed as a part of this study, so reporting with ARRIVE guidelines was not required. The joints were wrapped in PBS soaked gauze and stored at -20 °C.

### MRI scanning

Prior to imaging, the knees were brought to room temperature and wrapped in fresh PBS soaked gauze. The rat (*n* = 10) and mouse (*n* = 10) knees were imaged while held in in full extension in a custom jig (approximately 20° of knee flexion in rodents) in a 9.4T Bruker MRI scanner (Biospec 94/20 AV USR equipped with B-GA12S HP gradient; Bruker Corporation, Billerica, MA) with ParaVision 360 V3.3 and a 15 mm volumetric coil (T20093v3, Bruker Corporation, Billerica, MA) for signal transmission and reception. The rat knees were imaged using a Bruker T1_FLASH_3D scan sequence with parameters: TR = 38 ms, TE = 4.42 ms, Flip angle = 10 degree, FOV = 16 mm × 16 mm × 16 mm, Matrix = 160 × 160 × 160, voxel size = 0.1 mm isotropic, acquisition bandwidth = 27,778 Hz, averages = 4, scanning time = 65 min. The mouse knees were imaged using a Bruker T1_FLASH_3D scan sequence with parameters: TR = 30 ms, TE = 4.652 ms, Flip angle = 10 degree, FOV = 12 mm × 12 mm × 12 mm, acquisition matrix = 120 × 120 × 120, interpolation = 2 for read, phase and slice direction, image size = 240 × 240 × 240, voxel size = 0.05 mm isotropic, acquisition bandwidth = 27,778 Hz, averages = 12, scanning time = 87 min. These scan sequences were developed to provide adequate resolution and signal-to-noise ratio, to visualize all boundaries of the musculoskeletal soft tissues of interest for morphometric analysis, and to be within a reasonable time frame to maintain image quality and whole joint and tissue biomechanical function.

### Image segmentation

**Fig. 5:**
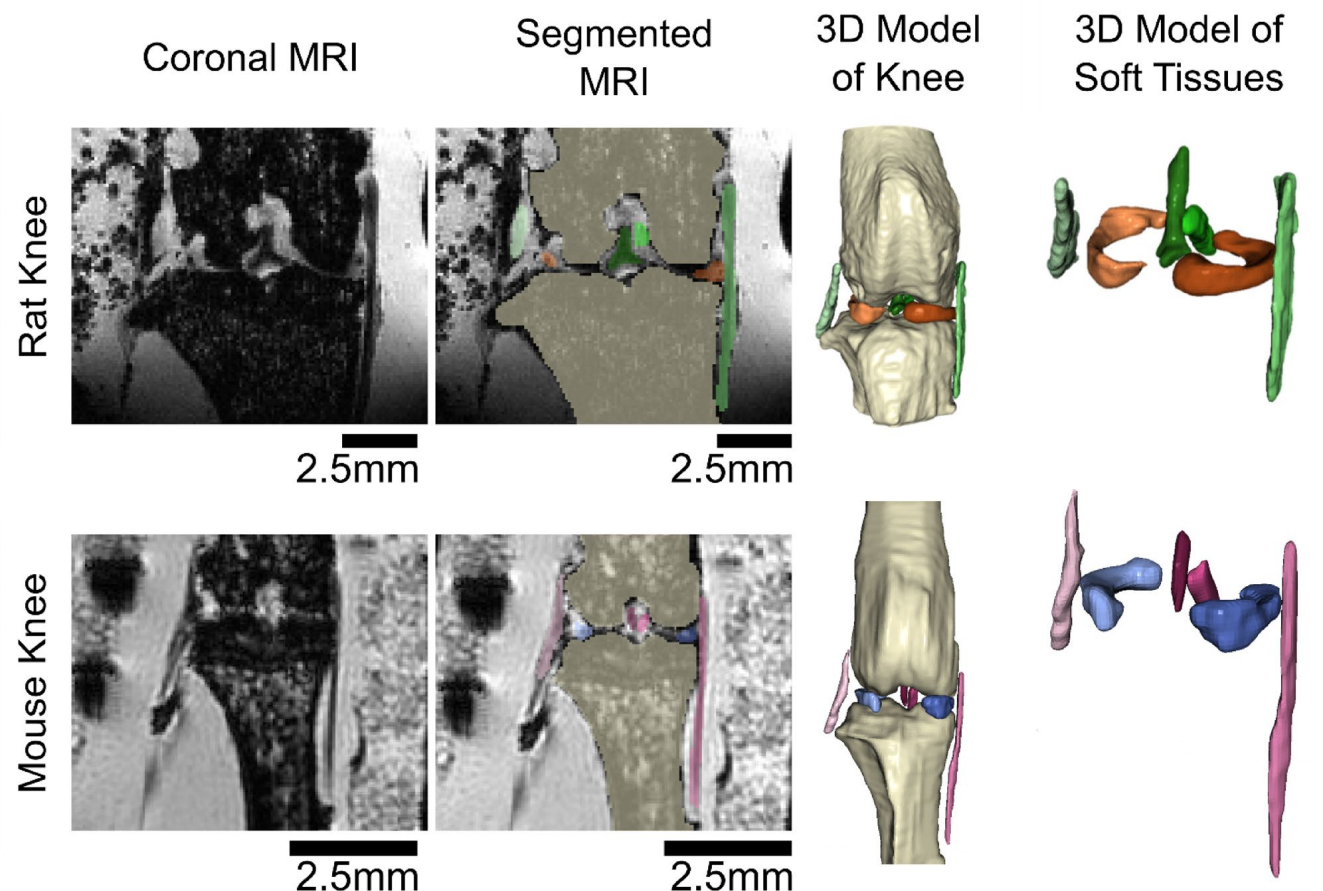
3D models of rat and mouse knees, created from segmented MRI scans. Images are of a mid-coronal slice of rat (20 week) and mouse (12 week) knees of similar skeletal maturity.

Following MRI scanning, images were imported into commercial software (Simpleware V-2024.06, Synopsis, Sunnyvale, CA) for manual segmentation. All image processing was performed by a single, trained viewer. Inter-repeater reliability was established on a subset of rat knee MRI scans. Separate segmentations were performed for six connective tissues per knee: the ACL, PCL, MCL, LCL, medial meniscus, and lateral meniscus (Fig. 5). Additionally, a single axial segmentation of the tibial bone was performed at the widest point of the tibial plateau in the axial view. The volume of the segmentation was divided by the height of the voxel to determine the area of the tibial plateau footprint for each knee. Following segmentation, 3D models of the connective tissues were generated, smoothed with a Gaussian filter (sigma = 1.0, isotropic voxels; Simpleware V-2024.06, Synopsis, Sunnyvale, CA), and exported as .stl files, using Simpleware and as previously described by Cone et al.^19^.

### Post-processing

**Fig. 6:**
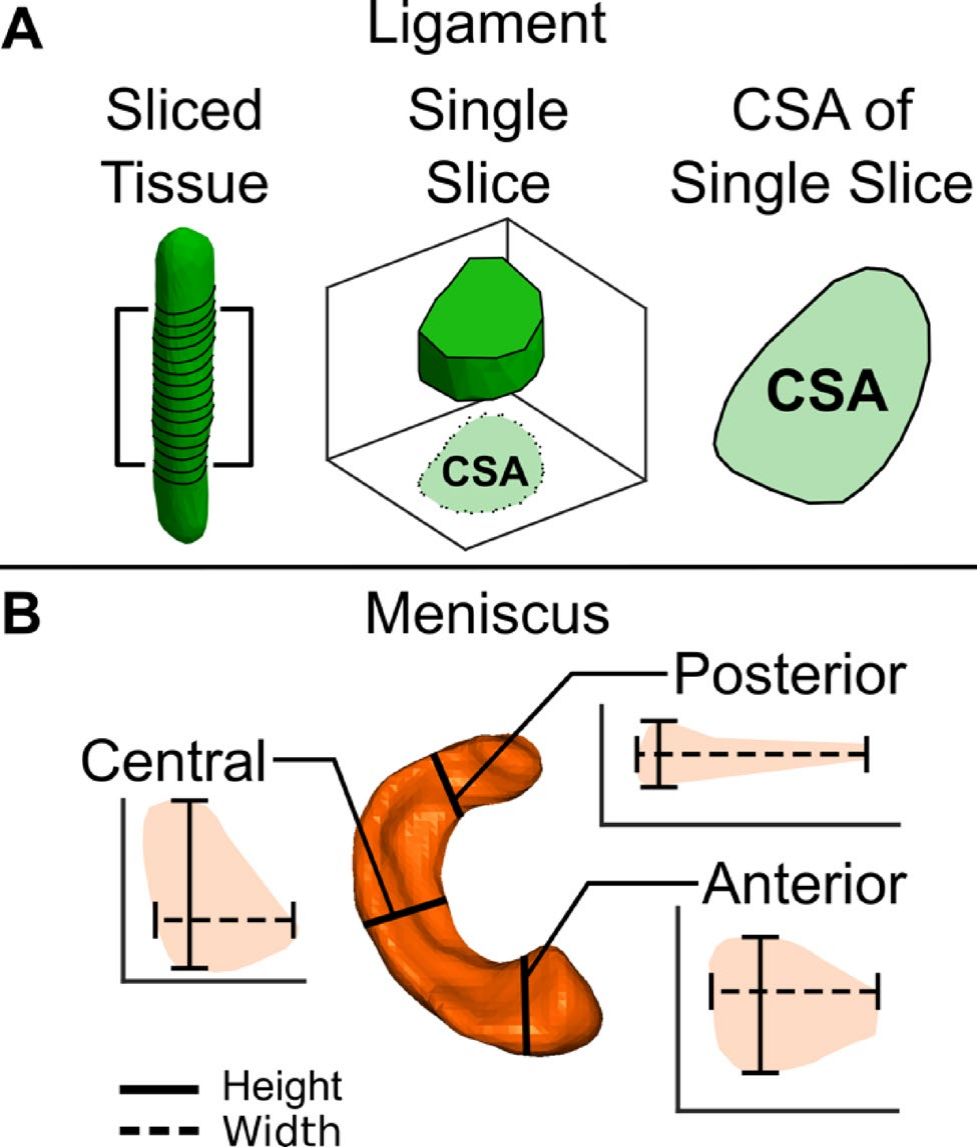
Morphometric properties of rat and mouse ligaments and menisci were recorded. (**A**) 3D ligament models were sliced in 0.1 and 0.05 mm increments for the rats and mice, respectively, and the CSA of each sliced was recorded. The average CSA of the middle 50% of each slice was recorded. (**B**) The 3D menisci models were sliced in the sagittal plane at the anterior and posterior horn and in the coronal plane at the central region. The maximum meniscal heights and widths from each region were recorded.

Using a custom MATLAB code, .stl files of all individual tissues were imported and converted into surface point clouds. Each ligament was rotated so that the longitudinal axis was aligned to a z-axis. The rat and mouse ligaments were then sectioned along the z-axis in 0.1 mm increments and 0.05 mm increments, respectively. Following slicing, each 0.1 mm–0.05 mm section was flattened onto a two-dimensional plane and the CSA was calculated (Fig. 6A). The CSA of the sections that composed the midsubstance, or middle 50%, of the ligament were averaged together to determine the CSA of that tissue. To avoid misinterpretation of the variability of the ligament morphometry near the bony insertion sites, the remaining portion of the ligament was excluded from further analysis. Each meniscus was rotated to an anatomic coordinate system and sliced in both the coronal and sagittal planes. The central region of the meniscus was determined as the middle three coronal plane slices (either the lateral-most or medial-most portion of the lateral and medial menisci, respectively), and the anterior and posterior regions were determined as the middle three sagittal plane slices on the anterior and posterior horn of each tissue. Meniscal height and width were measured in each central, anterior, and posterior region as ten equally spaced measurements of meniscus heights and widths across each tissue slice (Fig. 6B). The heights and widths of three slices from the anterior horn and posterior horn were averaged together to determine the height and width of the tissue in the two respective regions.^47,48^.

### µCT image validation

Prior to imaging the contralateral mouse knee joints (*n* = 10) were disarticulated and all soft tissues were removed from the tibiae. Throughout scanning, tibiae were kept hydrated in PBS soaked gauze. µCT scanning was performed at an 8 μm voxel size, an energy of 85,000 V, and an intensity of 47 µA with a Brüker SkyScan 1276 Micro-CT machine (Bruker Corporation, Billerica, MA). Whole tibiae were scanned to measure the CSA of the tibial plateau for validation of the MRI imaging and analysis technique. µCT images were reconstructed with Bruker MicroCT 3D.SUITE software that included NRecon, Dataviewer, and CTan (Bruker Corporation, Billerica, MA). Following reconstruction, the tibial plateau region was identified as the largest region on the tibia and that image was used for analysis. For each image, the bone was traced using FIJI/ImageJ and the area of that trace was calculated as the CSA.^49^.

### Statistical analysis

All statistical analysis was performed in R Studio (R Version 4.3.3, R. Posit Software, PBC, Boston, MA). All data was checked for normality using Shapiro-Wilk tests and checked for homogeneity of variances using Bartlett’s Tests. To compare average CSA of each ligament, the Kruskal-Wallis test was used as a non-parametric one way omnibus test followed by post hoc Tukey’s tests when significance was found. To compare meniscal heights and widths within anatomical regions and between medial and lateral menisci an aligned rank transform (ART) 2-way mixed non-parametric omnibus test was used followed by post hoc Wilcox’s rank sum tests when significance was found. To validate MRI imaging and analysis methodology a Wilcox’s rank sum test was performed to compare MRI measured tibial plateau CSA to microCT measured tibial plateau CSA. A Bland-Altman plot was created to determine proportional bias between the two techniques. To validate inter-repeater reliability a single score, two-way ICC agreement coefficient was calculated on a subset of rat ligament CSAs. Statistical results were corrected for multiple comparisons using Bonferroni corrections. Significance was set at α = 0.05.

## Data Availability

The datasets used and/or analyzed during the current study are available from the corresponding author on reasonable request.
